# Importance of Proprioceptive Information for Postural Control in Children with Strabismus before and after Strabismus Surgery

**DOI:** 10.3389/fnsys.2016.00067

**Published:** 2016-09-06

**Authors:** Maria P. Bucci, Hayette Soufi, Philippe Villeneuve, Lucile Colleville, Emmanuel Bui-Quoc, Cynthia Lions

**Affiliations:** ^1^Institut National de la Santé et de la Recherche Médicale- Université Paris 7, Robert Debré University HospitalUMR 1141, Paris, France; ^2^Vestibular and Oculomotor Evaluation Unit, ENT Department, Robert Debré University HospitalParis, France; ^3^Institut de PosturologieParis, France; ^4^Ophthalmology Unit, Robert Debré University HospitalParis, France

**Keywords:** children, postural control, strabismus, foam pad, proprioception

## Abstract

The objective of this study is to examine the role of proprioception in postural balance in children with strabismus before and after realignment of their visual axes by eye surgery. Postural recordings were made with the TechnoConcept® force platform in 23 children. Several conditions were studied, whether the subjects had both eyes open, or either the dominant or the non-dominant eye open, without and with foam pads of 4 mm underfoot. Recordings were performed before and after strabismus surgery. The surface area, the length and the mean speed of the center of pressure (CoP) were analyzed. Before strabismus surgery, all children showed better stability with both eyes open with respect to the condition with the non-dominant eye open; furthermore postural stability improved in the presence of foam pads. After surgery, the surface area of CoP decreased significantly, especially in the non-dominant eye viewing condition. We suggest that strabismic children use mainly proprioceptive information in order to control their posture, but also visual inputs, which are important for obtaining a good postural stability. The alignment of the visual axes after surgery provides enhanced postural stability, suggesting, again the major role of visual inputs in the control of posture. Proprioceptive plasticity after strabismus surgery may allow better visual rehabilitation.

## Introduction

Strabismus occurs in about 2% of children. Its origins are multiple, either sensory or motor, and result in a misalignment of the visual axis. The visual capabilities of strabismic children depends on the age of its apparition and also depends on the type of strabismus. A key factor in the diagnosis and treatment of strabismus is binocular vision, whether it is normal or abnormal and whether it is possible or not to restore it, depending on whether the strabismus occurs early or late. Indeed, early onset strabismus, which occurs within the first 2 years of life does not allow the establishment of binocular vision, and thus stereoscopic vision. When binocular vision is lacking, it implies that the subject only uses one eye. If strabismus occurs later in life, a normal development of binocular vision is possible. Strabismus surgery is often necessary to obtain ocular realignment, which is compulsory to restore stereoscopy and a better visual perception (von Noorden and Campos, 2006).

Posture is defined as the position of the various segments of the body, relative to each other and relative to the environment at a given instant (Paillard, 1976). The posture ensures first an antigravity role (Massion, 1997). Posture is the basis for movements. Postural adjustments ensure the support, the guidance and the coordination of gesture and movement. Postural control is defined as maintaining a given positional configuration of the body, on the basis of sensory systems information. It allows a recovery of the original configuration of the body after a loss of balance, through appropriate precise adjustments (Paillard, 1971). In humans, the reference posture can be defined as the state of the system when the subject stands without moving, with his or her feet parallel, without external forces other than gravity influencing his or her body. Several sensory information allows the maintenance of the posture, mainly the information from the vestibular, the visual, and the somatosensory systems (Nashner, 1976; Horak, 2010). The integration between these sensory informations occur via the central nervous system (CNS) which coordinates those informations and continuously generates the appropriate muscle responses to the inputs it analyses (Ivanenko et al., 1999). A disturbance of the sensory inputs causes unbalance, and a failure from one system can be compensated by sensory inputs from other systems (Brandt, 2003).

Several authors have shown that strabismic children are less stable than non strabismic age-matched children. In 2006, Matsuo et al. recorded the displacement of the CoP in 28 children with strabismus from 3 to 12 years old (13 of them with binocular vision) (Matsuo et al., 2006). These authors found that in children without binocular vision the length and the speed of the CoP displacement were larger than in children with binocular vision independently to the condition whether eyes were open or closed. These results suggested the important influence of binocular vision on postural control. In 2011, Legrand et al. recorded the surface and the speed of the CoP displacement in nine strabismic children from 4 to 8 years old. They showed that the postural parameters were significantly better when children had their eyes open with respect to a condition when eyes were closed (Legrand et al., 2011). In 2014, Lions et al. studied the role of proprioceptive information on postural control in strabismic children (Lions et al., 2014). They recorded the displacement of the CoP in 12 strabismic children from 4 to 10 years and compared these results to those obtained in 12 non strabismic age-matched children. Postural capabilities were recorded under both Romberg and Tandem condition with or without foam (15 cm of thickness). They found that postural parameters were significantly larger in strabismic children, particularly in Tandem position compared to the Romberg position, and on a foam surface. These authors hypothesized that strabismic children use more proprioceptive information to control their stability than non strabismic age-matched children.

Few studies have focused on the postural control after realignment of the eyes by oculomotor surgery. In 2006, Matsuo et al. found a significant increase in all the postural parameters (surface, length, speed) under both eyes open and eyes closed condition, after eye surgery. In contrast, Legrand et al. (2011) reported that 2 months after eye surgery postural performances improved significantly, in a small number of children though. These authors suggested that adaptive mechanisms could lead to improve postural control after strabismus surgery.

Based on these findings, the first objective of the present study is to examine the postural control of strabismic children and to further explore the role of the proprioceptive and visual informations. In order to achieve our goal, we placed foam soles under the feet of the children we tested, in several viewing condition (with both eyes open or under monocular viewing conditions, either with the dominant eye open or the non-dominant eye open). Our second objective is to evaluate the effect of eye surgery on posture. Therefore, recordings were also performed after surgery.

Our hypotheses are as follows: (1) postural instability will increase when proprioceptive information is disrupted; (2) postural stability will differ depending on the viewing eye; (3) postural control will improve after surgery.

## Materials and methods

### Subjects

Twenty-three strabismic children between 4.6 and 14.8 years old (mean age: 8.4 ± 0.87 years) participated in the study. Strabismic children were recruited from the Department of Ophthalmology, Robert Debré Children's Hospital in Paris. All subjects underwent ophthalmologic and orthoptic evaluation.

The investigation adhered to the principles of the Declaration of Helsinski and was approved by our institutional Human Experimentation Committee (*Comité de Protection des Personnes, CPP Ile de France V, Hôpital Saint-Antoine*). Written consent was obtained from the children's parents after an explanation of the experimental procedure.

### Ophtalmologic and orthoptic examination

All strabismic children underwent ophthalmologic and orthoptic examination to evaluate their visual function. The Table 1 shows clinical data of each child before and after strabismus surgery.

**Table 1:** **Clinical characteristic of strabismic children before and after surgery**.

**Children(years)**	**Before surgery**				**After surgery**			
	**Glasses correction**	**Corrected visual acuity**	**Angle of strabismus (prism D)**	**Stereoacuity (TNO)**	**Type of strabismus**	**Type of surgery**	**Angle of strabismus (prism D)**	**Stereo acuity (TNO)**
C1 (4.6)	RE: +7.75	RE: 20/20	55E'T + 5H'DT	–	Early onset esotropia	a (RE)	2E'	–
	LE: +7.50	LE: 20/20	50 ET + 3 HDT				6XT	
C2 (4.8)	RE: +4.75 (−1.5) 20°	RE: 20/20	40 E'T	–	Early onset Esotropia			
	LE: +4.50 (−1.00) 165°	LE: 20/20	40 ET					
C3 (5.4)	RE: +1.75 (−2.00) 85°	RE: 20/20	50XX'T	60″	Intermittent exotropia	b (RE)	25XX'T	60″
	LE: +1.00 (−1.00) 100°	LE: 20/20	25XXT				16XXT	
C4 (5.4)	RE: +1.25 (−0.50) 5°	RE: 20/20	8 XX'T	60″	Exotropia	b (LE)	8X'	–
	LE:+ 1.50	LE: 20/20	45 XT + HDT				35XXT + 8HDT	
C5 (5.7)	RE: +1 (−0.50) 50°	RE: 20/20	25 XX'T	60″	Intermittent exotropia	b (LE)	4X'	60″
	LE: +0.50	LE: 20/20	35 XXT				6X	
C6 (5.9)	RE: +3.00 (−1.00) 60°	RE: 20/20	45E't + 10 H'GT	–	Early onset esotropia	a (LE)	40E'T	–
	LE: +3.75 (−1.25) 10°	LE: 20/20	40 ET				16ET	
C7(5.9)	RE: +2.50 (−0.50) 15°	RE: 20/20	30 E'T	–	Early onset Esotropia			
	LE: +3.25 (−1.25) 150°	LE: 20/20	4 ET					
	ADD +2.50							
C8 (6.2)	RE: +1.75 (−1.25) 84°	RE: 20/50	55 E'T	–	Early onset Esotropia			
	LE: +1.75 (−1.00) 56°	LE: 20/63	55 ET					
C9 (6.4)	RE: +2.75 (−0.50) 180°	RE: 20/20	55 E'T + 3HDT	–	Early onset esotropia	a (RE)	4E'T	200″
	LE: +3.75 (−0.50) 160°	LE: 20/20	60 ET				2ET	
C10 (7.0)	RE: 0.00	RE: 20/20	4X'	120″	Intermittent exotropia	b (RE)	4E'	120″
	LE: 0.00	LE: 20/20	30XXT + 6 HTD				6X	
C11 (7.4)	RE: +1.25 (−1.50) 5°	RE: 20/20	4E'	–	Intermittent exotropia	b (LE)	20E'T	–
	LE: +1.25 (−1.75) 175°	LE: 20/20	25XT+2HGT				6XT	
C12 (7.6)	RE: +5.00 (−0.75) 145	RE: 20/25	35 E'T	–	Early onset esotropia	c (RE and LE)	4X'T	–
	LE: + 6.25 (−2.00) 180	LE: 20/20	12 ET				10XT	
C13 (7.7)	RE: +3.5 (−2.50) 175°	RE: 20/20	20X'T +H'TD	–	Exotropia			
	LE:+3.75 (−2.50)175°	LE: 20/20	20XT+3HTD					
C14 (8.5)	RE: +0.5 (−1.75) 175°	RE: 20/20	25 XX'T	120″	Intermittent exotropia			
	LE: −0.5 (−0.25) 170°	LE: 20/20	14 XXT					
C15 (9.6)	RE: −0.25	RE: 20/20	25 XX'T	60″	Intermittent exotropia			
	LE: −0.25	LE: 20/20	18XXT+ 4 HDT					
C16 (9.9)	RE: +1.50 (−1.50) 0°	RE: 20/20	0′	120″	Intermittent exotropia	b (RE)	25XX'T	15″
	LE: +1.25 (−1.25) 175°	LE: 20/20	10XT				16XT	
C17 (10.1)	RE: +0.50 (−1.00) 100°	RE: 20/20	6X'	60″	Intermittent exotropia	b (RE)	6X'	60″
	LE: −0.25 (−0.50) 170°	LE: 20/20	8XXT				8XXT	
C18 (10.6)	RE: +1.25 (−1.00) 10°	RE: 20/20	45 X'T+5 H'GT	–	Intermittent exotropia	b (LE)	16 XX'T	60″
	LE: +1.25 (−1.00) 175°	LE: 20/20	35 XT				18XT + 4 HGT	
C19 (10.9)	RE: (−0.50) 160°	RE: 20/20	25 XX'T	60″	Intermittent exotropia	b (LE)	2E'	30″
	LE: (−0.75) 180°	LE: 20/20	25 XXT				2X	
C20 (13.1)	RE: +0.50 (−1.00)175°	RE: 20/20	30XX'T	200″	Intermittent exotropia			
	LE: +1.00 (−1.25) 5°	LE: 20/20	30 XXT					
C21 (14.2)	RE: +4.50 (−1.75) 90°	RE: 20/25	20 E'T	–	Esotropia acquired	a (RE)	4E'T	240″
	LE: +2.50 (−1.25) 10°	LE: 20/20	16 ET				4ET	
C22 (14.6)	RE: (−0.50) 80°	RE: 20/20	35 E'T	–	Early onset Esotropia	a (RE)	16 E'T	–
	LE: (−0.50) 90°	LE: 20/20	25 ET				10 ET + 2HDT	
C23 (14.8)	RE: +4.50	RE: 20/20	50 E'T	–	Early onset esotropia	a (LE)	20E'T	–
	LE: +6.00 (−2.25) 130°	LE: 20/50	50 ET				8ET	

*Child age, glasses correction, corrected visual acuity, angle of strabismus before and after surgery, non-squint eye, stereoacuity before and after surgery and type of strabismus. The deviation of the eyes was assessed with cover-uncover test and prism; the binocular vision was evaluated with the TNO test for stereoscopic depth discrimination. LE, left eye; RE, right eye. X XT and X'-X'T, intermittent exotropia measured at far distance (5 m) and at near distance (30 cm) respectively. ET and E'T, esotropia measured at far (5 m) and at near (30 cm) distance, respectively. HT, hypertropia measured at far distance (5 m). For the type of surgery: a: Tightening of the medial rectus muscle and resection of the lateral rectus muscle, b: Tightening of the lateral rectus muscle and resection of the medial rectus muscle, and c: Cuppers technique (faden procedure)*.

The visual acuity was measured for each eye separately at far distance (5 m) with the Monoyer chart (an optometric chart containing 10 rows of letters, each row corresponding to 1/10 visual acuity). The type and the angle of strabismus (i.e., the manifest deviation of one eye) was measured at near (33 cm) and far distance (5 m) using a base-in and a base-out prism with a Berens prism bar. Stereoacuity threshold based on disparity detection was evaluated with the TNO random dot test for stereoscopic depth discrimination.

The monocular visual acuity varied between 20/63 and 20/20. Nine children (C1, C2, C6-9, C12 C22-23) had early onset esotropia (i.e., esotropia which began before the age of 2 years old), one child (C21) had acquired esotropia (i.e., esotropia which began after the age of 2 years old), 11 children (C3, C5, C10, C11, 14–20) had intermittent exotropia, and two children (C4 and C13) had acquired exotropia. Among these children, eight (C3–5, C10, C14-17, C19, and C20) had a binocular vision (between 120″and 60″ s of arc), and the other 15 children had no binocular vision.

Sixteen of these subjects have been examined 2–6 months after surgery (see Table 1). Note that it is well known that eye surgery alters the proprioceptive information of the operated eye (Steinbach and Smith, 1981) and at least 2 months are needed in order to be sure that proprioceptive receptors in the extra-ocular muscle are not any more influenced by surgery (Buisseret, 1995). All children but one (C16) had a decrease of the squint angle. Among these children, nine (C3, C5, C9, C10, C16-21) children had binocular vision after surgery (between 240 and 15 s of arc). In five children, stereoscopic vision improved (C16, C19) or was restored. (C9, C18, C21). Three types of surgery were performed: Tightening of the medial rectus muscle and recession of the lateral rectus muscle: (a), tightening of the lateral rectus muscle and recession of the medial rectus muscle: (b), and Cuppers technique (faden procedure): (c).

### Material

Postural performance was measured by a platform (AFP40/16 Stabilotest, principle of strain gauge) consisting of two dynamometric clogs (Standards by the *Association Française de Posturologie*, produced by TechnoConcept®, Céreste, France). During, 25.6 s the excursions of the center of pressure (CoP) were measured. The surface of the CoP was calculated following Gagey's standards (Gagey et al., 1993; Gagey and Weber, 1999). The equipment included a 16-bit analog-digital converter and the acquisition frequency was 40 Hz.

### Postural recording test

The examination took place in a dark room. The children stood on the platform in the Romberg position, their heels being placed 4 cm apart and their feet positioned symmetrically with respect to the participant's sagittal axis at a 30° angle. A large dark curtain was suspended from the ceiling to form a semi cylindrical black space around each child in order to avoid any visible visual scene around the screen. A clown's red nose located 200 cm at the eye level in front of them had to be fixated. Children had to stay as still as possible, with the arms along the body and had to be careful to avoid any stiff.

### Visual and postural conditions

Postural control was recorded under three visual conditions: binocular eye viewing with both eyes open (BEV), or monocular eye viewing with the dominant eye (DEV) or with the non-dominant squinting eye (NDEV). Two sensorial conditions were studied: without or with a foam pad (+F) between the platform and the feet, in order to change the cutaneous inputs from the foot sole. Therefore, a 4 mm-thick foam was placed on the platform's footprints (see Supplementary Figure). For each of the visual condition three postural recordings were taken successively. The order of the visual conditions was randomly chosen.

### Data processing

To quantify the effect of visual sensorial conditions on the postural performance, several postural parameters were analyzed: the surface area (mm^2^), the length (mm), and the mean speed (mm/s) of the CoP. Note that all these postural parameters are used to evaluate postural capabilities in humans and a decrease of such parameters suggested an improvement in postural stability A.F.P., 1984; Gagey et al., 1993.

The surface area and the length of the CoP measures the its spatial variability. The surface of the CoP corresponds to an ellipse with 90% of CoP excursions. The length of CoP is the path of the CoP. These two postural parameters are uncorrelated; indeed, the inner surface of the same length can be different (Vuillerme et al., 2008).

The mean speed of the CoP represents a good index of the amount of neuro-muscular activity required to regulate postural control (Geurts et al., 1993).

### Statistical analysis

An ANOVA was firstly performed for comparing the two different groups of children with convergent and divergent strabismus but it failed to show any statistical difference between the two groups of children. Consequently postural data reported below will be showed for children with convergent and divergent strabismus together. For all children, analysis of variance ANOVA with repeated measures was used to compare postural data in the three different visual conditions and in the two postural conditions (with and without foam) and the Greenhouse-Geisser correction was applied. The *post-hoc* Bonferroni was also performed. For the explore the eventual surgery effect, a similar ANOVA was with 3 × 2 × 2 (vision, foam, and surgery) factorial within-subject analysis was applied. The effect of a factor was considered as significant when the *p*-value was below 0.05.

## Results

**Figure 1:**
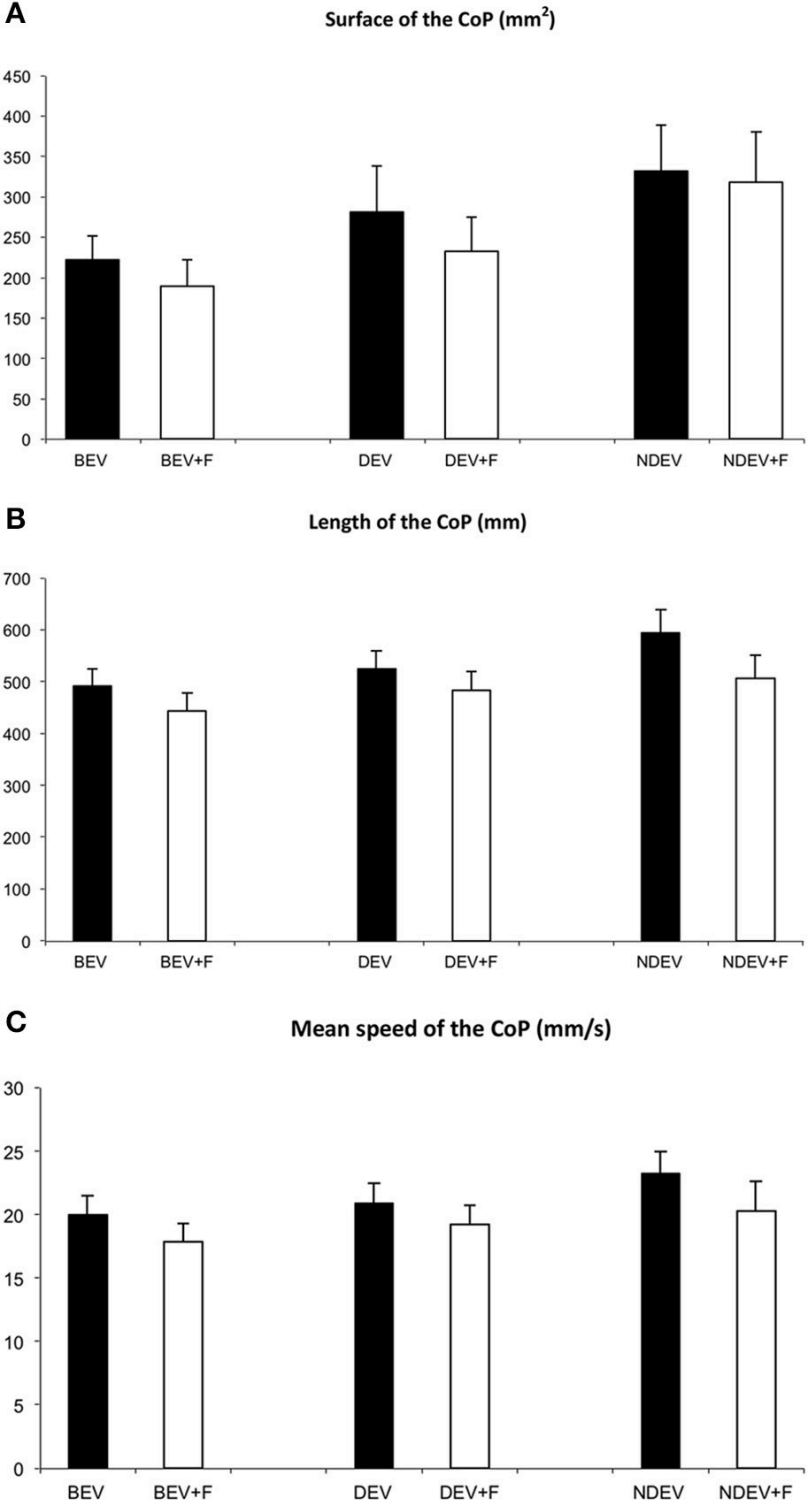
**Mean surface area (mm^**2**^) (A), length (mm) (B), and mean speed (mm/s) (C) of the CoP in all strabismic children tested under the three visual conditions: binocular eye viewing (BEV), monocular eye viewing with dominant eye (DEV) and with non-dominant, squint eye (NDEV) on the two sensorial conditions: without and with Foam pad (+F)**. Vertical bars indicate the standard error.

Figure 1A shows the mean surface of the CoP during the three visual conditions and the two sensorial conditions in strabismic children. The ANOVA test showed a significant effect of vision [*F*_(2, 44)_ = 5.63, *p* < 0.007]; indeed *post-hoc* comparison showed that the mean surface of the CoP was significantly smaller under binocular viewing condition with respect to the monocular viewing NDEV condition (*p* < 0.005). ANOVA showed also a significant effect of sensorial condition [*F*_(1, 22)_ = 5.91, *p* < 0.02]; indeed the mean surface of the CoP was significantly smaller in the +F condition.

Figure 1B shows the mean length of the CoP in the three visual conditions and both sensorial conditions in strabismic children. The ANOVA test showed a significant effect of vision [*F*_(2, 44)_ = 6.09, *p* < 0.005]; indeed *post-hoc* comparison showed that the length of the CoP was significantly smaller under binocular viewing condition with respect to the monocular viewing NDEV condition (*p* < 0.003). ANOVA showed also a significant effect of sensorial condition [*F*_(1, 22)_ = 4.63, *p* < 0.04], indeed, the mean length of the CoP was significantly smaller in the +F condition.

Figure 1C shows the mean speed of the CoP during the three visual conditions and in both sensorial conditions tested in strabismic children. The ANOVA test showed a significant effect of vision [*F*_(2, 44)_ = 3.81, *p* < 0.04]; indeed *post-hoc* comparison showed that the mean speed of the CoP was significantly smaller under binocular viewing condition with respect to the monocular viewing NDEV condition (*p* < 0.02). ANOVA showed also a significant effect of sensorial condition [*F*_(1, 22)_ = 4.72 *p* < 0.04]; indeed the mean speed of the CoP was significantly smaller in the +F condition.

**Figure 2:**
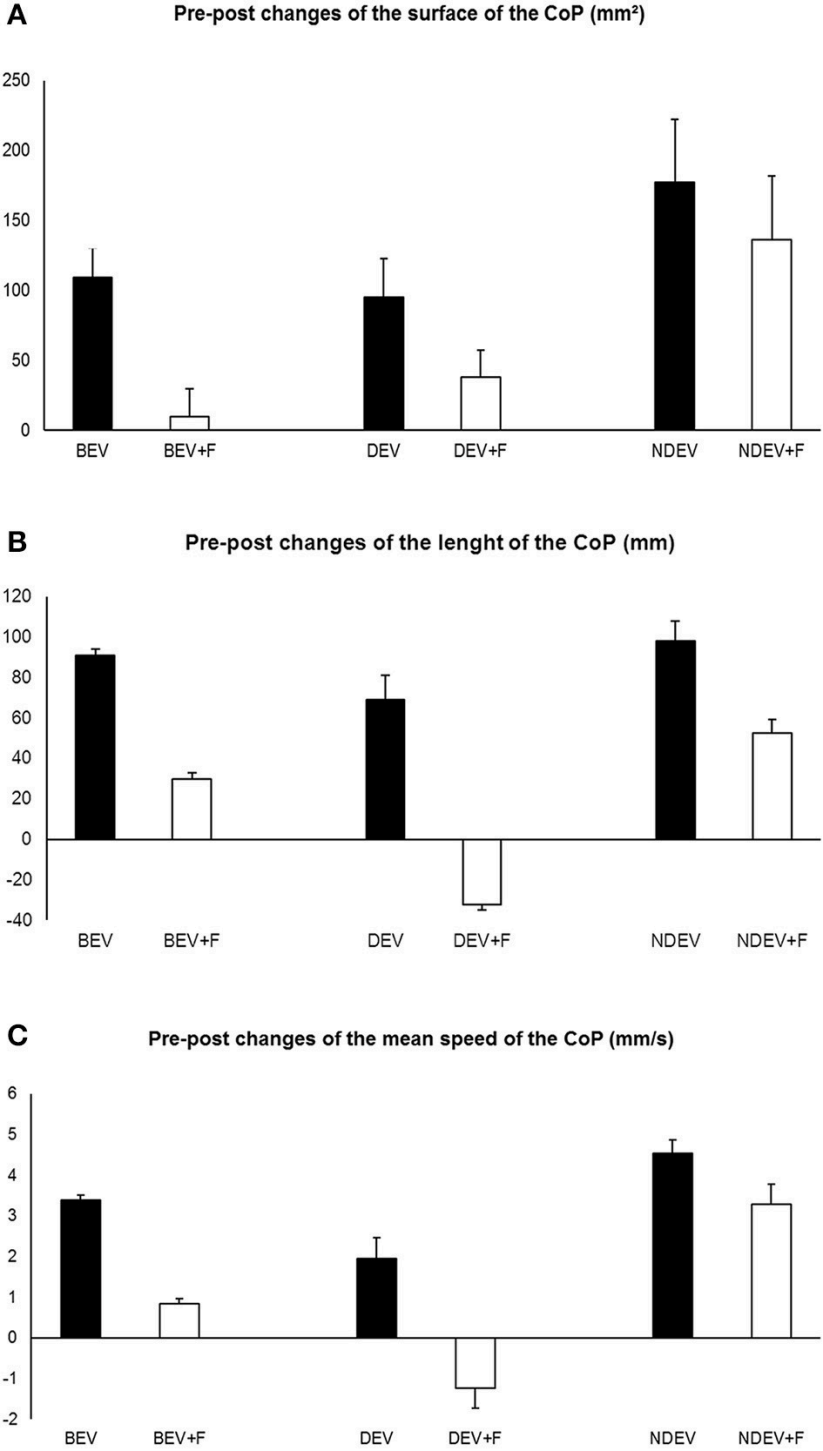
*****Pre-post*** eye surgery changes of the surface area (A), of the length (B), and of the mean speed (C) of the CoP in 16 children tested under the three visual conditions: binocular eye viewing (BEV), monocular eye viewing with dominant eye (DEV) and non-dominant, squint eye (NDEV) on two postural conditions: without and with Foam pad (+F)**. Positive values indicate a decrease of the value after strabismus surgery. Vertical bars indicate the standard error.

Figure 2A shows the changes of the surface area of the CoP in the three visual conditions and in both sensory conditions in the 16 children tested before and after strabismus surgery. In all conditions the surface area of the CoP decreased after surgery. The ANOVA test reported a significant effect of surgery only [*F*_(1, 15)_ = 5.77 *p* < 0.003]: the surface area of the CoP was smaller after surgery.

Figure 2B shows the changes of the length of the CoP in the three visual conditions and both sensory conditions in the 16 children tested before after strabismus surgery. In all but one condition (dominant eye viewing with foam pad, DEV, F+) the length of the CoP became shorter after surgery. The ANOVA test showed a significant effect of vision only [*F*_(2, 30)_ = 4.23 *p* < 0.03]; the *post-hoc* comparison showed that the length of the CoP was significantly smaller under binocular viewing condition with respect to the monocular viewing DEV condition (*p* < 0.02).

Figure 2C shows changes of the mean speed of the CoP in the three visual conditions and in the two sensory conditions in the 16 children tested before and after strabismus surgery. In all but one condition (dominant eye viewing with foam pad, DEV, F+) the mean speed of the CoP became smaller after surgery. The ANOVA test showed a significant effect of vision only [*F*_(2, 30)_ = 7.58 *p* < 0.03]; the *post-hoc* comparison showed that the mean velocity of the CoP was significantly smaller under binocular viewing condition with respect to the other two monocular viewings condition (*p* < 0.001 and *p* < 0.05 for DEV and NDEV condition, respectively).

## Discussion

The main findings of this study are as follows: (i) Before strabismus surgery, strabismic children showed better stability with both eyes open with respect to the condition when the non-dominant eye is open; (ii) Postural stability improved in the presence of foam pads; (iii) After surgery, postural control improved significantly. These findings are discussed individually below.

### Postural stability is better when both eyes are open

This study reported that all postural parameters examined were smaller when both eyes were open with respect to the monocular condition when the non-dominant eye is open. This finding is in line with previous studies showing the important role of visual input, particularly for children, to control their posture (Forssberg and Nashner, 1982). Furthermore, our data showed also that a poor visual input such as the information from the non-dominant eye seems to be not good enough to obtain a good posture stability. A recent study from Moraes et al. (personal communication) reported in healthy young adults the different roles of binocular visual informations with respect to monocular ones, in the task of sensory reweighting the postural stability, when a subject adapts to changes in his or her environment. Based on this study we suggest that binocular informations are important to obtain postural stability of the body.

### Foam pad improved postural stability

The present study shows that thin foam of 4 mm improved postural body sway; note that this result contrasts our previous hypothesis. Recall that sensory information from the plantar surface of the foot is important for postural control (Allum et al., 1998; Maurer et al., 2001); for instance, force, pressure, and support surface qualities informations are encoded by plantar cutaneous afferents that are strictly linked to the CNS in order to reach body stability (Meyer et al., 2004). In the present study, we reported that all postural parameters examined (the surface, the length, and the mean speed of the CoP) decreased significantly when children were on a 4 mm foam pad suggesting that such fine foam pad is able to improve proprioception activity in strabismic children leading to better postural control. This result is only apparently in contrast with those found in the study of Lions et al. (2014) showing that postural stability in strabismic children was more impaired when they were tested on a soft foam. In fact the foam pad used in this former study was 15 mm thick, in contrast to the foam pad used in the present study which was 4 mm thick. Indeed, as showed by Patel et al. (2008), the effect of the foam pad on postural stability depends on the foam properties. As suggested by Assländer and Peterka (2014), adult healthy subjects are able to reweight their sensory systems in order to control their stability. Children with strabismus could also be able via adaptive mechanisms to use proprioceptive information in order to ensure a better postural stability. This suggests the important role of the cutaneous foot sole input in the postural control of strabismic children. This new result brings a significant clinical perspective. Recall that currently the treatment of strabismus is based on optical correction, treatment of amblyopia and realignment of eye axes by surgery (which by the way could affect the proprioceptive information from the eyes). It would be possible to add a therapeutical tool to better manage strabismic children if we could modify the proprioceptive system of the body, along with treating the visual system. It should be noted that, at least in France, clinician used very frequently foot sole in order to stimulate plantar sensory input and improve postural stability in children with neuro-developmental disorders (Martins da Cunhà and Alves da Silva, 1986). We could make the hypothesis that children with strabismus, in order to compensate their deficit of visual inputs, could take advantage from postural reeducation that stimulates other sensorial inputs (vestibular and/or proprioceptive). Further studies on such issue need to be done in order to confirm this hypothesis.

### Strabismus surgery improves postural stability

Our data showed that strabismus surgery improved postural control, particularly for the surface area of the CoP. We found also that when children were viewed with their non-dominant eye postural stability had tended to improve. This is a quit logic and expected result because the non-dominant eye was the deviated eye and visual input from this eye was poor before surgery. This finding reinforces the hypothesis that strabismus surgery improved visual inputs and extra-ocular proprioceptive inputs simultaneously. Both sensory and motor components of the eyes are improved by surgery and the better quality of visual inputs can be responsible for better postural stability.

## Limitations

A larger number of children with convergent and divergent strabismus will be necessary in order to explore further the role of the squint eye on postural control. Also, a group of healthy non-strabismic children need to be tested in order explore whether the improvement of foam pad in postural control is also observed in non-strabismic children.

## Conclusion

In conclusion, this study highlights important results concerning the role of proprioceptive inputs on postural control in strabismic children. For the first time, we showed that a thin foam pad of 4 mm improved postural stability in strabismic children. Also, we found that strabismus surgery improved postural stability. It would be interesting to study the evolution of postural control after eye movement's therapy, to further explore the role of extra-ocular proprioceptive inputs on postural control, and develop new rehabilitation tools in strabismus.

## Author contributions

MB, conception of the study, drafting the work, final approval of the version to be published; HS, analyzed data, final approval of the version to be published; PV, interpretation of data, final approval of the version to be published; EB, selected patients, final approval of the version to be published; LC, analyzed data, final approval of the version to be published; CL, drafting the work, final approval of the version to be published.

## Funding

Study was supported by *Fondation pour la Recherche Médicale* “Pathophysiology of the Visual System 2013” (DVS20131228511).

### Conflict of interest statement

The authors declare that the research was conducted in the absence of any commercial or financial relationships that could be construed as a potential conflict of interest.
