# Supplementary figures and images for: Importance of Proprioceptive Information for Postural Control in Children with Strabismus before and after Strabismus Surgery
Source: Front Syst Neurosci. 2016 Sep 6;10:67. doi: 10.3389/fnsys.2016.00067 (PMC5012200; doi:10.3389/fnsys.2016.00067)

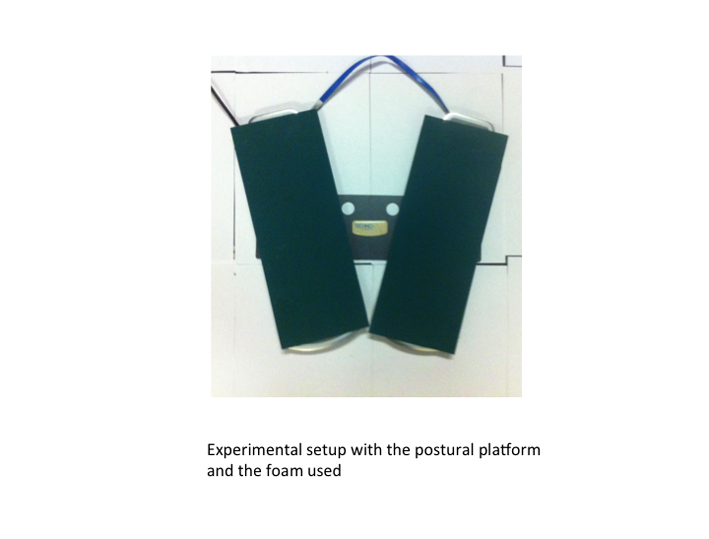

Supplement: Supplementary file 1 [file Image1.TIFF]
